# Breast Desmoid Tumor After Bilateral Breast Reduction: A Case Report

**DOI:** 10.7759/cureus.92240

**Published:** 2025-09-13

**Authors:** Hannah Grace Talbot, Emily Hecox, Jared M Davis

**Affiliations:** 1 Surgery, University of Mississippi School of Medicine, Jackson, USA; 2 Plastic Surgery, University of Mississippi Medical Center, Jackson, USA

**Keywords:** breast benign and malignant surgery, breast plastic surgery, breast reduction surgery (brs), extra-abdominal desmoid tumor, oncoplastic breast surgery, plastic and reconstructive surgery

## Abstract

Desmoid tumors are rare soft tissue tumors that can occur at various anatomic sites. Often, patients present with symptoms that are secondary to either external compression or local invasion into nearby structures. While desmoid tumors are rare in the breast, a significant portion of breast desmoid tumors occur in women who have undergone prior breast surgery.

We detail a case report of a 60-year-old woman who presented with an enlarging, painful breast mass in the lower outer quadrant of the right breast two years after undergoing bilateral breast reduction for symptomatic macromastia. Furthermore, we reviewed the literature for the treatment of breast desmoid tumors.

The patient underwent diagnostic imaging, biopsy, and multidisciplinary care. Ultimately, she opted for wide local excision, and the margins on permanent pathology were negative with the closest margin being 5mm. With negative margins, no adjuvant radiation was done. She continues to be followed for surveillance and has no clinical or radiologic signs of recurrence at one year after resection.

Breast desmoid tumors are rare and often associated with prior breast surgery. Patients who have undergone elective breast surgery may present to any range of medical providers with a firm breast mass that may or may not be symptomatic. Diagnostic imaging should be pursued to rule out malignancy rather than assume pathology such as fat necrosis. The principles of oncoplastic breast surgery can be used to achieve functional and aesthetic results in the context of wide local excision.

## Introduction

Desmoid tumors are rare mesenchymal neoplasms characterized by infiltrative growth and propensity for recurrence that have an estimated incidence of 3-5 cases per million person years, accounting for roughly 0.2% of breast tumors [1,2]. Also referred to as aggressive or deep fibromatosis, they do not tend to metastasize but can be associated with significant morbidity. Symptoms are often secondary to external compression on vascular or nervous structures and vary depending on the anatomic site of the tumor [3]. Specific to the breasts, the tumors are often associated with a history of breast trauma or surgery [4], with a prior retrospective study finding 44% of patients with breast desmoid tumors had a history of breast surgery and 6% had undergone prior breast reduction [5]. Among that cohort, desmoid tumors occurred after breast augmentation, partial mastectomy, mastectomy without reconstruction, and mastectomy with implant or autologous reconstruction [5]. These tumors have also been associated with pregnancy and familial adenomatous polyposis (FAP) and defects in the adenomatous polyposis coli (APC) gene [4]. Breast desmoid tumors are of special concern because they can clinically mimic breast carcinoma and impact quality of life, often presenting as a palpable, firm mass that may have associated pain or attachment to the chest wall [2,4-8]. While the majority of breast masses are benign and pain is not usually a presenting symptom of breast cancer [9,10], physicians who perform breast surgery, including breast reduction, and others who treat patients who have undergone breast surgery should be aware of desmoid tumors as part of the differential diagnosis and be well-versed in their treatment. 

Recently, nirogacestat became the first United States Food and Drug Administration (FDA)-approved systemic therapy for symptomatic, progressing desmoid tumors [11]. Traditionally, surgical resection with a wide margin was the mainstay of treatment, although what gross margin is adequate remains a topic of debate due to the infiltrative nature of the tumors and tendency for local recurrence [7,12]. In recent years, active surveillance has gained prevalence for desmoid tumors that are stable in size and not symptomatic. For tumors that are enlarging, symptomatic, or non-resectable, antihormonal agents such as selective estrogen receptor modulators, tyrosine kinase inhibitors, and chemotherapeutic agents have been used for treatment with mixed efficacy [1,3,4,6,8,13]. Due to the rare nature of the disease, there is a lack of breast-specific treatment guidelines [4]. However, it is recommended that patients with desmoid tumors receive multidisciplinary care and give consideration to screening for FAP [10,14]. Herein, we present a case of a desmoid tumor localized to the right breast after bilateral breast reduction and the treatment approach. 

## Case presentation

A 60-year-old woman underwent bilateral breast reduction with nipple areolar complex preservation on a superomedial pedicle with a Wise-pattern skin reduction. The reduction specimens weighed 1015g on the right and 1120g on the left, and pathology demonstrated benign breast tissue bilaterally and usual ductal hyperplasia on the right. Her immediate post-operative course was uneventful. Two years after surgery, she presented to her primary physician with a painful, enlarging lower outer quadrant mass of the right breast. Besides her history of breast reduction, she had no other history of breast trauma, surgery, or biopsy. She had undergone routine colonoscopy with a single tubular adenoma six months prior to the diagnosis of desmoid tumor. Family history was significant for an older sister with metastatic breast cancer. She was referred for diagnostic imaging, including mammogram and ultrasound. 

**Figure 1:**
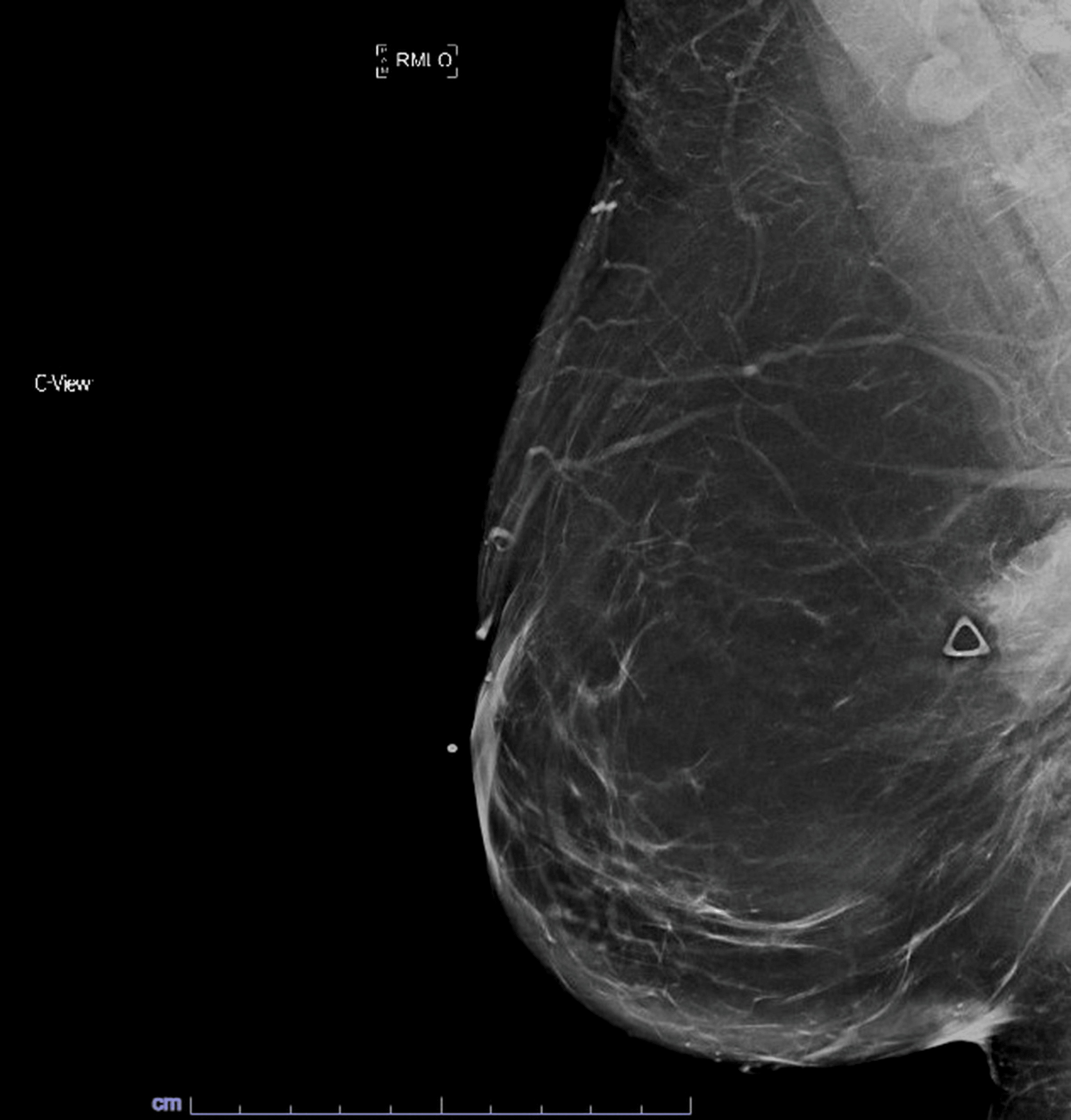
Right mediolateral oblique mammogram This figure shows the mediolateral oblique view of the patient's diagnostic mammogram that was done after she presented with a palpable breast mass.  The mass was better visualized on this view than on the craniocaudal compression view.

**Figure 2:**
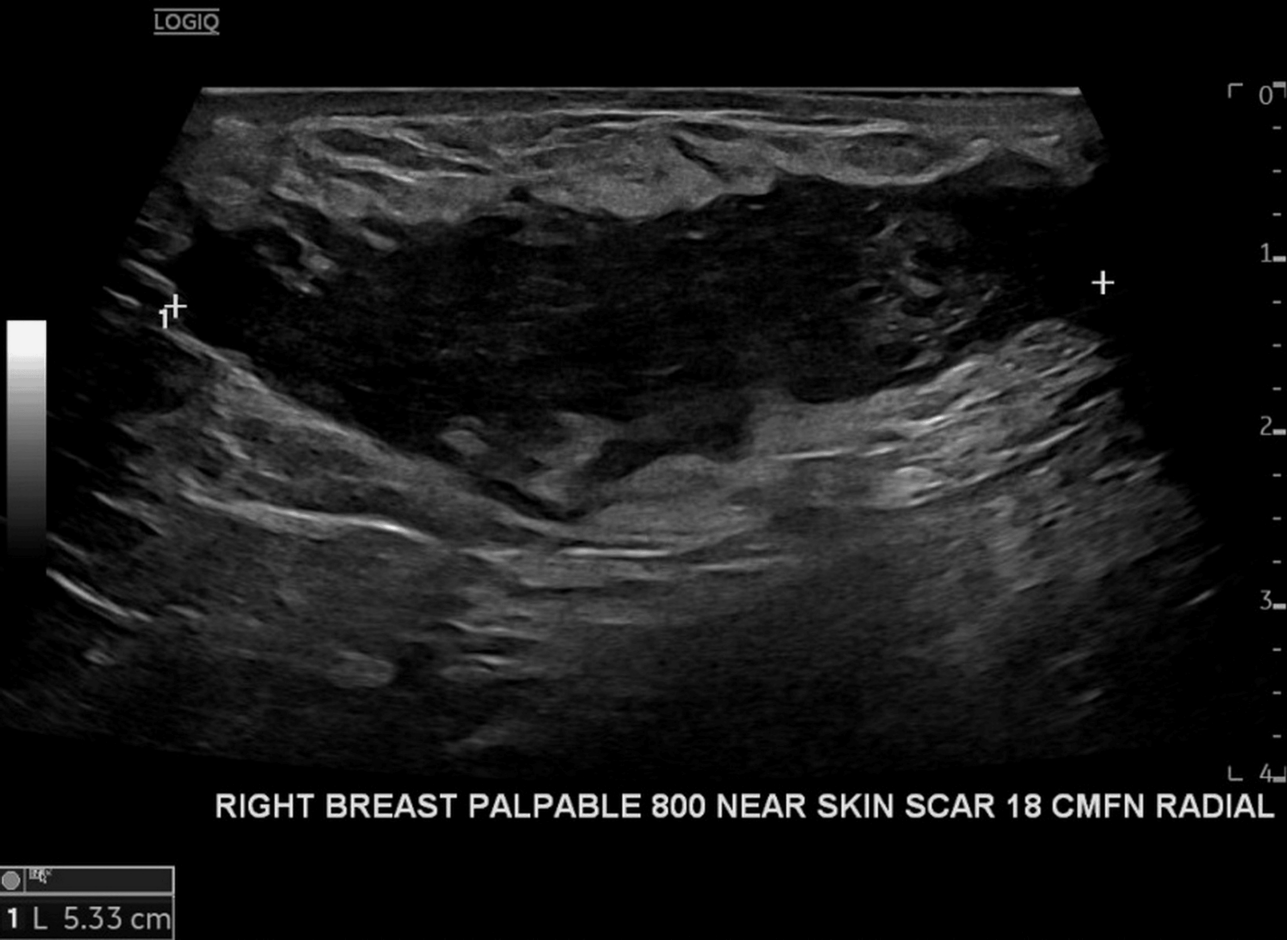
Right breast ultrasound This ultrasound view of the right breast mass at 8 o'clock demonstrates palpable breast mass

Her mammogram (Figure 1) demonstrated a mass in the right breast lower outer quadrant measuring over 4cm and was interpreted as BIRADS-3, probably benign. Ultrasound (Figure 2) demonstrated an elongated, hypoechoic mass measuring 6.0x2.6x1.7cm with indistinct margins approximately 18cm from the nipple in the 8:00 o’clock position of her right breast. Subsequent ultrasound-guided biopsy revealed benign fibroblastic proliferation of spindle cells with focal areas of infiltration into thin vasculature and nearby tissues and widespread beta-catenin nuclear positivity, most consistent with desmoid-type fibromatosis.

The patient was referred back to the operating surgeon from the breast reduction for evaluation. On exam, the palpable mass was noted to be deep to the lateral aspect of the horizontal limb incision from the Wise-pattern breast reduction and was freely mobile relative to the chest wall. No axillary lymphadenopathy was present.

Due to the exam findings and her family history, a genetic evaluation was ordered. Her multidisciplinary care also includes radiation oncology evaluation to discuss the potential role of adjuvant radiation. After a detailed discussion, she opted for wide local excision. The consent discussion focused on the pathology and pathophysiology of desmoid tumors, the tendency for local recurrence, the importance of symptoms to decision making, and the role of medical management and radiation therapy. We also discussed the potential aesthetic implications of re-operative surgery.

**Figure 3:**
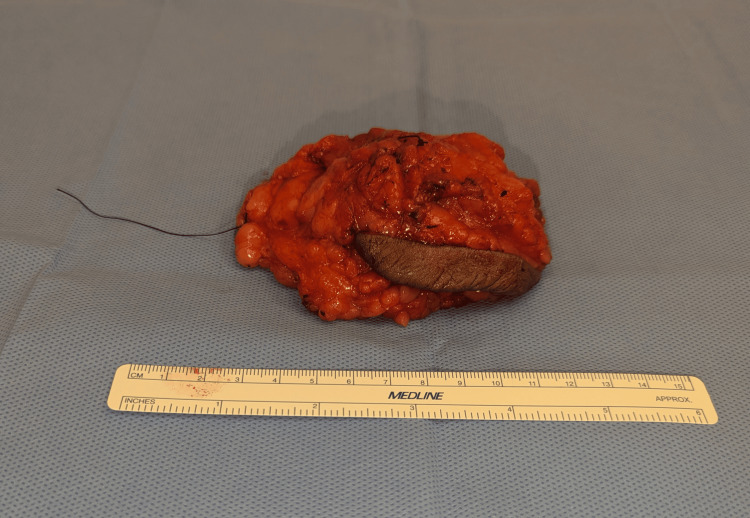
The resected specimen This representative image shows the relative size of the specimen. The skin is centered around the lateral aspect of the horizontal incision from the Wise-pattern breast reduction. Note the sutures for orientation, with the long stitch being lateral and the short stitch marking the superior margin. Also note the presence of normal-appearing tissue at the periphery of the resected specimen.

**Figure 4:**
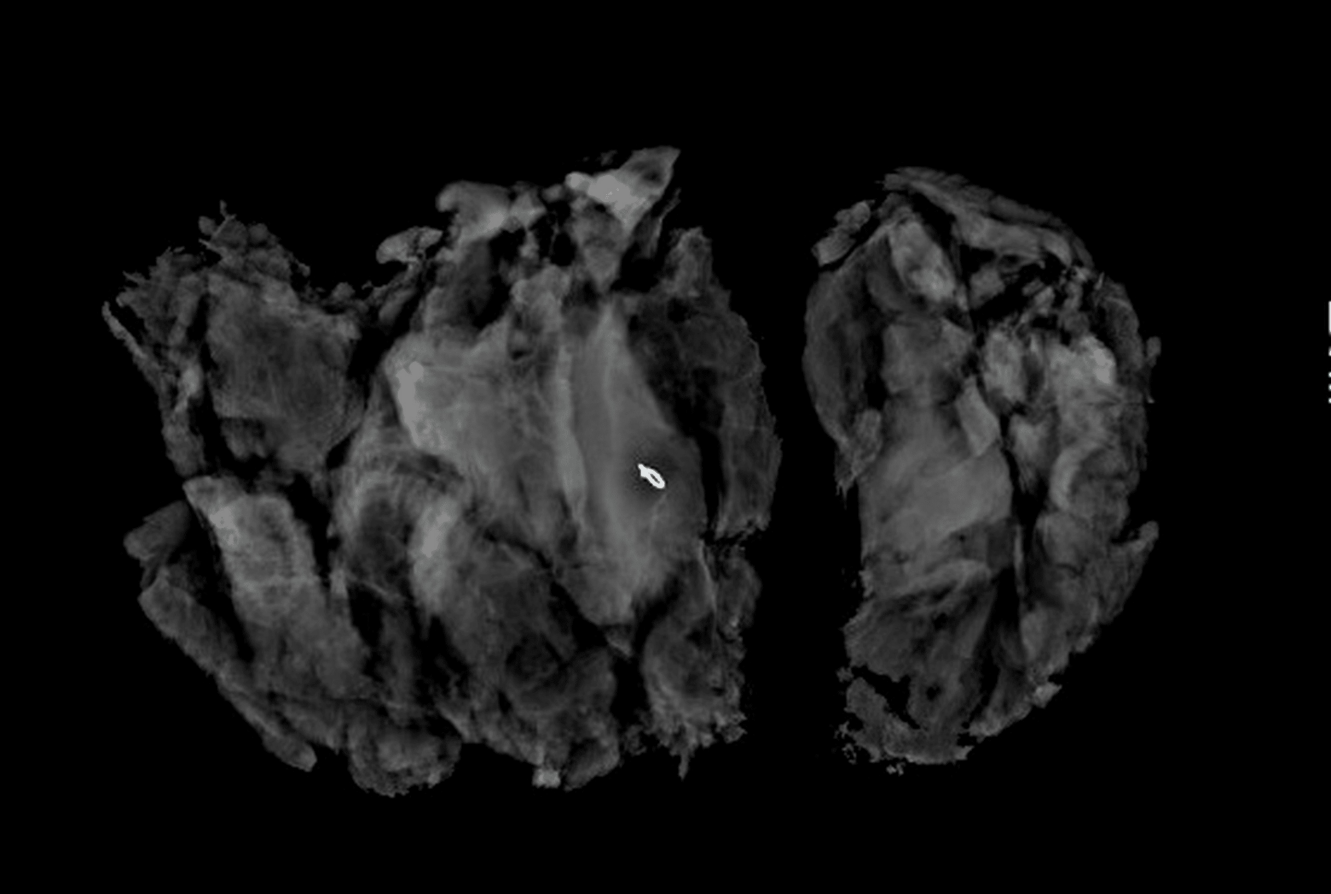
Longitudinal specimen mammogram See the specimen mammogram containing the clip from the core needle biopsy. In this image, the specimen has been split by the pathologist.

**Figure 5:**
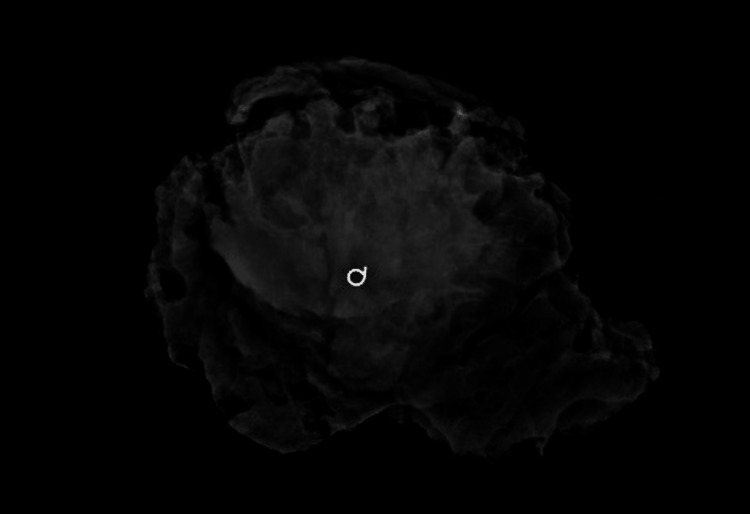
Axial view of specimen mammogram This axial specimen mammogram is an orthogonal view to the one in Figure 4 and demonstrates the clip centered within the specimen.

**Figure 6:**
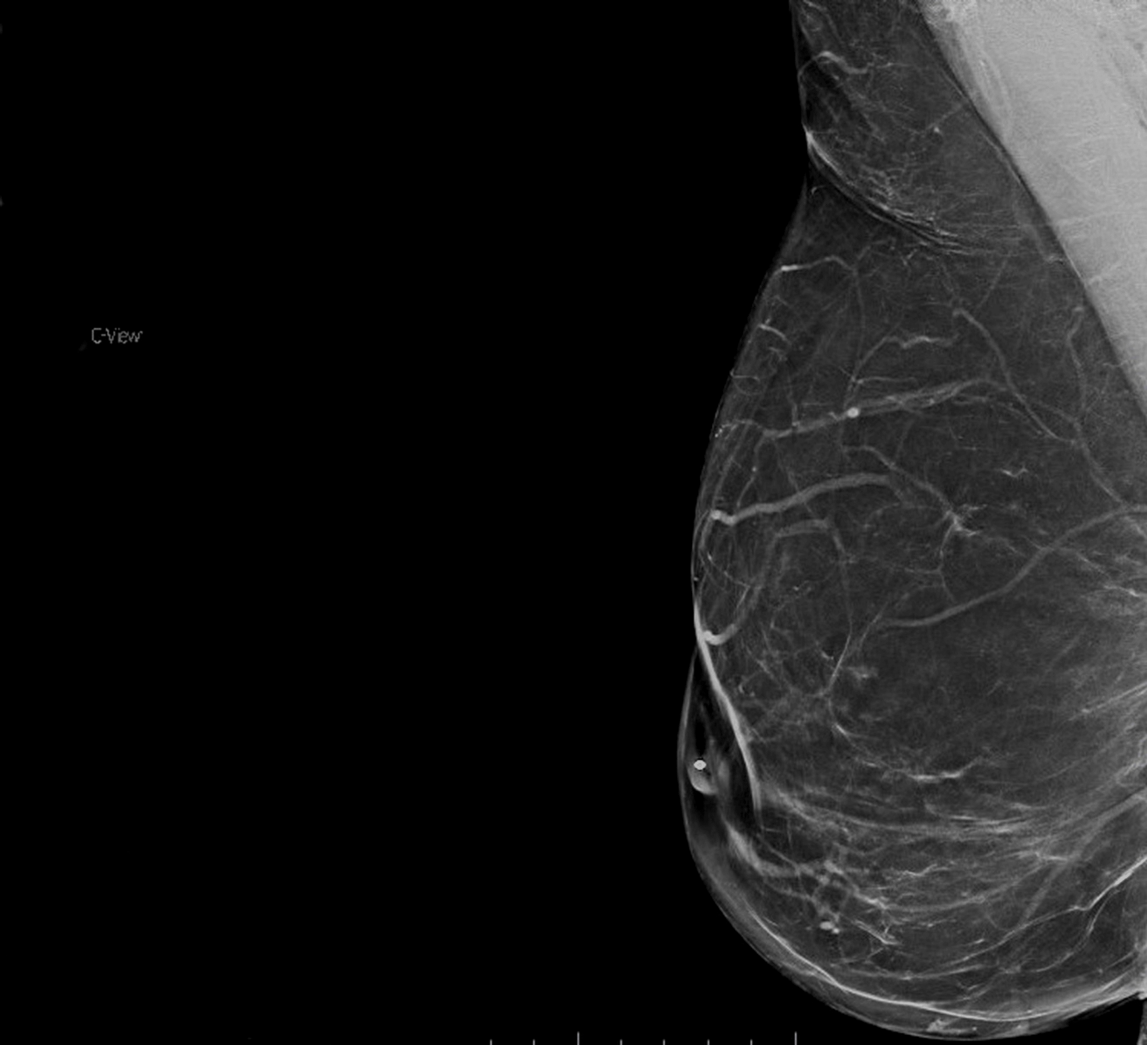
Post-operative mediolateral oblique mammogram This view demonstrates the post-operative mammogram. Note the absence of the desmoid tumor visualized in Figure 1.

The operation was done under general anesthesia and was done in a fashion to preserve cosmesis and not increase the scar burden, by planning the excision to incorporate the lateral aspect of the horizontal incision along with the underlying mass. A fusiform incision was made, including the lateral aspect of the horizontal limb of the Wise pattern breast reduction. The cutaneous scar, surrounding skin, and the underlying mass were excised down to the chest wall, aiming for a 1 cm margin in all dimensions. The specimen was oriented and for permanent pathology. The resected mass measured 11.5 x 8.5 x 4 cm (Figure 3). Specimen mammograms with orthogonal views demonstrated the biopsy clip centered in the specimen (Figure 4 and Figure 5). Histologic examination was significant for 139g specimen containing a 4.8cm well-circumscribed desmoid tumor with negative margins and the nearest being 5 mm at the superior margin. After discussion of the risks and benefits of adjuvant radiation therapy in the setting of negative margins, she opted to defer adjuvant radiation. At one year after resection, she is recurrence free and is being followed with mammogram and clinical examinations every six months. The mammogram one year after resection can be seen in Figure 6. 

## Discussion

Desmoid tumors are rare tumors; however, when they occur in the breast, there is often an association with prior breast surgery [4,5]. There remains a relative paucity of reports of desmoid tumors presenting after breast reduction surgery. While there are no guidelines specific to breast desmoids, the NCCN guidelines provide adequate guidance for appropriate multidisciplinary care [15]. Appropriate workup would include imaging and core needle biopsy to rule out malignancy. Management should be based on symptoms, with asymptomatic tumors that are size-stable being considered for active surveillance, including clinical exam and imaging at initial three-month intervals to determine if the desmoid demonstrates progression [4]. Asymptomatic tumors that do not progress may continue to be followed, and symptomatic, enlarging, or distressing tumors should be considered for systemic medical therapy or surgical excision. Recently, nirogacestat became the first FDA-approved systemic therapy for symptomatic desmoids [11]. Should a patient demonstrate progression on this or other systemic therapy, and the tumor is resectable, they should be considered for wide local excision with a goal of negative margins and consideration of adjuvant radiation for positive margins [4]. Adjuvant radiation in the context of positive margins has been shown to have no significant difference in 10-year recurrence rates compared to surgical excision with negative margins [12]. In anatomic locations that are functionally or aesthetically sensitive, the principles of oncoplastic breast surgery can be used to optimize form and function while practicing sound oncologic principles [16] and adjuvant therapy can still be beneficial.

## Conclusions

This case demonstrates that surgical excision remains a viable option for the treatment of biopsy-proven breast desmoid tumors. Treatment priorities should be to rule out malignancy, appropriately triage patients to active surveillance, systemic therapy, or surgical excision, achieve negative margins when possible using basic oncoplastic techniques to preserve form and function, and to employ adjuvant radiation to reduce the risks of treatment failure in the context of positive margins. Our patient’s desire for surgical excision was gained through shared decision-making and multidisciplinary care.
